# A Lung Adenocarcinoma Patient With a Rare *EGFR* E709_T710delinsD Mutation Showed a Good Response to Afatinib Treatment: A Case Report and Literature Review

**DOI:** 10.3389/fonc.2021.700345

**Published:** 2021-06-11

**Authors:** Yu Wei, Yueli Cui, Yao Guo, Lei Li, Liang Zeng

**Affiliations:** ^1^ Department of Respiratory and Critical Care Medicine, Jiangsu Provincial Hospital of Traditional Chinese Medicine, Nanjing, China; ^2^ Department of Research and Development, Nanjing Geneseeq Technology Inc., Nanjing, China; ^3^ Department of Radiology, Jiangsu Provincial Hospital of Traditional Chinese Medicine, Nanjing, China

**Keywords:** NSCLC, *EGFR* mutation, exon 18, E709_T710insdelD, afatinib

## Abstract

For advanced lung adenocarcinoma patients with common epidermal growth factor receptor (*EGFR*) mutations (exon 19 deletions or the exon 21 L858R mutation), tyrosine kinase inhibitors (TKIs) are the standard therapies, and achieve favorable responses. However, for the rare *EGFR* deletion-insertion mutation of exon 18, there is no evidence of the efficacy of EGFR TKIs. Herein, we report a lung adenocarcinoma patient harboring a rare *EGFR* E709_T710delinsD mutation who was treated with afatinib as the first-line therapy and achieved a progression-free survival of 23 months. After the disease progressed, the patient received almonertinib treatment and exhibited a stable disease. This case indicated that non-small cell lung cancer patients harboring the *EGFR* E709_T710delinsD mutation could benefit from afatinib treatment, followed with almonertinib treatment, as a potential therapeutic strategy.

## Highlights

- A lung adenocarcinoma patient harbored a rare exon 18 deletion-insertion mutation of in *EGFR* (E709_T710delinsD) at with a mutant allele frequency of 74.8%.
- The patient was treated with afatinib as the first-line therapy and achieved a progression-free survival of 23 months.
- After the disease progressed, almonertinib was administered as the second-line therapy for the NSCLC patient, and led to a stable disease.

## Introduction

Lung cancer, of which 80% - 85% is classified as non-small-cell lung cancer (NSCLC), has the highest death rate of all cancers worldwide (1, 2). Somatic activating mutations in the epidermal growth factor receptor (*EGFR*) are the most common oncogenic driver mutations in Asian NSCLC patients, with a prevalence of 47% (3). Such mutations typically occur within exons 18 - 21. The most common *EGFR* mutations (nearly 85% - 90%) in NSCLC patients are deletions in exon 19 (19Del) and the L858R point mutation in exon 21, which are defined as classical mutations. The remaining 10% - 15% of *EGFR* mutations are non-classical mutations, including point mutations and deletions in exon 18, and point mutations and insertion mutations in exon 20 (4).

For NSCLC patients with *EGFR* mutations, it has been well documented that patients with *EGFR* 19Del and L858R mutations exhibit good clinical responses to EGFR tyrosine kinase inhibitors (TKIs). Clinically, first-line therapy with EGFR TKIs is recommended and significantly improves the survival of NSCLC patients with *EGFR* variations (5, 6). Although the T790M mutation in exon 20 is resistant to first- and second-generation EGFR TKIs, it is responsive to the third-generation EGFR TKI, osimertinib (7). However, there is insufficient clinical evidence to confirm the sensitivity of exon 18 mutations to EGFR TKIs. In this case, we report a lung adenocarcinoma patient with a rare *EGFR* exon 18 deletion-insertion mutation (E709_T710delinsD) that responded well to afatinib and achieved a progressive-free survival of 23 months. Following the development of afatinib resistance, the patient then benefited from almonertinib treatment.

## Case Presentation

**Figure 1:**
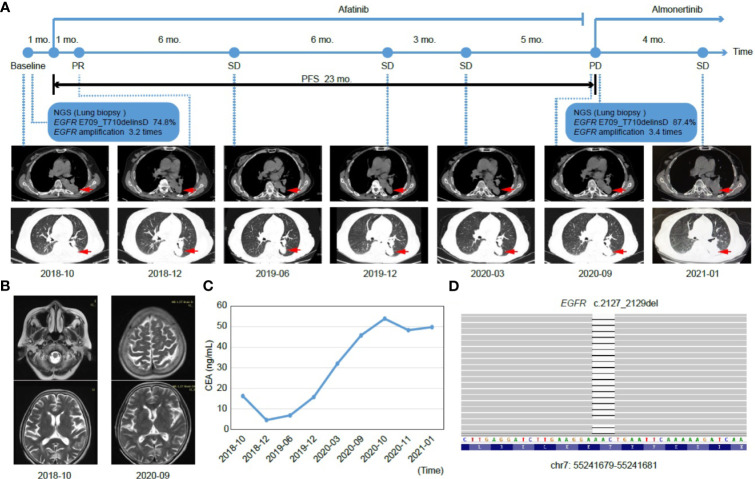
Tumor progression of the patient before and after treatment. **(A)** The timeline of therapies and tumor progression are indicated (Top). CT images revealed lesions in lower left lung. The tumor is indicated by red arrows. PFS, progression-free survival; PR, partial response; SD, stable disease; PD, progressive disease; NGS, next-generation sequencing; mo., months. **(B)** Brain MRI scans revealed no metastasis before (October 2018) and after (September 2020) treatment. **(C)** Line chart showing changes in the levels of the serum tumor biomarker, CEA (carcinoembryonic antigen) during the course of treatment. **(D)** The EGFR p.E709_T710delinsD mutation was visualized by IGV software. Deletions are indicated with a black dash (–).

A 70-year-old Chinese female non-smoker with no family history of cancer suffered from repeated coughing for more than 10 days in October 2018. Computed tomography (CT) of the chest revealed a 5.1 × 3.6 cm density mass in the dorsal segment of the lower left lung (**Figure 1A**). Magnetic resonance imaging (MRI) scans showed that there were no metastases in the brain (**Figure 1B**). The level of the serum tumor biomarker, carcinoembryonic antigen (CEA), was 16.27 ng/mL, which was much higher than the normal range of < 5.0 ng/mL (**Figure 1C**). Immunohistochemical analyses revealed that the tumor cells of the left lung were positive for thyroid transcription factor-1 (TTF-1) and Napsin A, whereas focal staining was positive for CK5/6 (data not shown). Based on those data, the patient was diagnosed with stage II lung adenocarcinoma (cT3N0M0 of TNM staging system), and was recommended surgical treatment according to the National Comprehensive Cancer Network Guidelines. However, the lung lesion was tightly connected to great vessels, and thus, could not be completely removed by surgery. To identify a more effective treatment, a left lung biopsy was subjected to genetic testing of fourteen lung cancer-related genes using next-generation sequencing (NGS). A rare *EGFR* exon 18 mutation (p.E709_T710delinsD) was identified with a mutant allele frequency (MAF) of 74.8%, and accompanied by *EGFR* amplification and a *TP53* p.M246_T256del mutation (**Table 1** and **Figure 1D**).

**Table 1:** Genetic alterations detected in lung cancers.

Genes	Alternations	Coding change	MAF	MAF
			10/2018	09/2020
*EGFR*	p.E709_T710delinsD	c.2127_2129del	74.8%	87.4%
*EGFR*	Amplification	/	3.2 times	3.4 times
*TP53*	p.M246_T256del	c.735_767del	26.5%	49.5%
*PIK3CA*	p.E545K	c.1633G>A	–	7.6%
*PIK3CA*	p.E542K	c.1624G>A	–	1.0%
*CHD8*	p.N761Kfs*11	c.2282dup	–	35.4%
*RB1*	Splice-site mutation c.861+2T>A	/	–	80.3%
*SMAD4*	Single copy loss	/	–	/
*NRG1*	p.T591M	c.1772C>T	–	1.2%
*TERT*	p.T249M	c.746C>T	–	0.6%
*TNFAIP3*	p.M181I	c.543G>C	–	12.7%
*UGT1A1*	p.L124F	c.372G>T	–	7.5%

-, not detected; /, not applicable; MAF, mutant allele frequency.

In consideration of the patient’s age and physical condition, the patient and her family refused radiotherapy and chemotherapy. Rather, the second-generation EGFR TKI, afatinib, was administered as a once-daily oral dose of 30 mg in November 2018. One month post treatment, a partial response (PR) was achieved with lesion shrinkage in the left lung according to the RECIST response criteria, and the serum CEA level returned to normal (4.58 ng/mL, **Figures 1A, C**). Seven months after afatinib treatment, the left lung tumor volume was further reduced, accompanied by a stable CEA level (6.87 ng/mL, **Figures 1A, C**). The lung lesion was re-evaluated using CT scans in December 2019 and revealed an increased tumor size (**Figure 1A**). The serum CEA level (15.80 ng/mL) was also elevated at that time (**Figure 1C**). However, the patient exhibited a stable disease (SD) according to RECIST response criteria. In March 2020, CT scans revealed no marked enlargement of the lesion, while the CEA level was markedly elevated (32.05 ng/mL, **Figures 1A, C**). In spite of the SD that was maintained during this period, the possibility of slow progress was not ruled out. In the case of remission and with no obvious adverse reactions, the patient continued to be treated with afatinib until September 2020. At that time, there was no metastasis in the brain, but the target lesion in the lower left lung increased with a concomitant increase in CEA levels (45.80 ng/mL), thus, indicating progressive disease (PD) (**Figure 1A–C**).

After the development of afatinib-resistance, another tumor biopsy was collected and subjected to NGS-based genetic testing of 425 cancer-related genes. The results revealed that the MAF of the *EGFR* E709_T710delinsD mutation increased from 74.8% to 87.4% (**Table 1**). The patient was then switched to a third-generation EGFR TKI, almonertinib, with a once-daily oral dose of 80 mg, as it was thought to impart fewer adverse effects than osimertinib. In January 2021, although the lesion in the left lung was slightly enlarged and serum CEA levels (49.81 ng/mL) were higher than those in September 2020 (**Figures 1A, C**), the patient was considered as SD under the RECIST response criteria. Until February 2021, the patient was still receiving almonertinib treatment.

## Discussion

Patients with *EGFR* mutations exhibit various responses to TKI treatment, and the classical mutations in exons 19 and 20 exhibit good responses to such treatments. However, the sensitivity of exon 18 mutations to TKIs has not been determined. According to the latest update of the Catalogue of Somatic Mutations in Cancer (COSMIC) database, the E709_T710delinsD mutation accounts for only 0.064% of *EGFR* mutations (17/26499), and thus, its clinical significance is unclear.

In recent years, studies have reported the responses of lung cancer patients with E709_T710delinsD mutations to EGFR TKIs (**Table 2**) (2, 8–16). Among patients receiving gefitinib or erlotinib, one achieved a PR, two achieved a SD, and nine others were non-responders (i.e., PD). These patients achieved PD and SD experienced a short PFS from 0.9 to 8 months, with a median of 3 months (2, 8–13).

**Table 2:** All reported cases of the *EGFR* E709_T710delinsD mutation in lung adenocarcinoma patients treated with EGFR TKIs.

Author, publication year	Age (years)/Gender	Smoker	Stage	Cancer Type	Treatment	Response to TKI (time)	PFS (months)	OS (months)
**First-generation EGFR TKIs**								
Wu et al. (8)	61/F	No	IV	ADC	Gefitinib	SD	5.1	79.0
	65/M	Yes	IV	ADC	Gefitinib	PD	0.9	11.1
Wu and Shih (9)	57/F	No	IV	ADC	Gefitinib	PD	6.0	24.1
	79/M	Yes	IV	ADC	Gefitinib	SD	6.2	6.2
	68/M	Yes	IV	ADC	Gefitinib	PD	2.3	29.5
Ackerman et al. (10)	88/F	No	IV	NSCLC	Erlotinib	PR (4 months)	NA	NA
Martin et al. (2)	60/M	No	IV	ADC	Erlotinib	PD	1.0	3.0
Isaksson et al. (11)	NA	NA	IV	NA	Erlotinib	PD	8.0	NA
Sousa et al. (12)	66/F	Yes	IV	ADC	Gefitinib	PD	3.0	24.0
	46/F	Former heavy	II	ADC	Erlotinib	PD	4.0	26.0
	57/F	No	IV	ADC	Erlotinib	PD	3.0	18.0
Klughammer et al. (13)	50/F	No	III or IV	NSCLC	Erlotinib	PD	1.3	1.7
**Second-generation EGFR TKIs**								
Kobayashi et al. (14)	63/M	NA	IV	ADC	Erlotinib	SD	NA	NA
					Afatinib	Tumor shrinkage (1 month)	NA	
Ibrahim et al. (15)	52/F	No	IV	ADC	Afatinib	2 months	NA	NA
D’Haene et al. (16)	57/F	No	III	ADC	Afatinib	PR (12 months); PD	12.0	36.0
Present case	70/F	No	II	NSCLC	Afatinib	PD	23.0	Ongoing
					almonertinib	SD	NA	

NA, data not-available; F, female; M, male; PFS, progression-free survival; OS, overall survival; TKI, tyrosine kinase inhibitor; ADC, lung adenocarcinoma; NSCLC, non-small cell lung cancer; PR, partial response; SD, stable disease; PD, progression disease.

Another group compared the efficacy of first-generation (gefitinib and erlotinib), second-generation (afatinib and dacomitinib, and neratinib), and third-generation TKIs (osimertinib and CO1686) *in vitro* (14), and identified that cells transfected with the E709_T710delinsD mutation were more sensitive to second-generation TKIs, and especially afatinib. That study also showed that a lung adenocarcinoma patient who acquired the E709_T710delinsD mutation benefited from afatinib after erlotinib treatment failed. Ibrahim et al. also reported a patient who had reduced lung nodules after 2 months of afatinib treatment (15). Similarly, a 57-year-old female with lung adenocarcinoma was treated with afatinib after the disease progressed following chemotherapy and maintained a PR for one year (16). In the current case, the patient was treated with afatinib and achieved a PFS of 23 months. After the development of afatinib-resistance, almonertinib was then administered and the patient achieved SD.

Otherwise, Zeng et al. found that the E709_T710delinsD mutation was an acquired drug resistance mechanism and not sensitivity to afatinib in an advanced lung adenocarcinoma patient with an *EGFR* exon 18 E709H mutation (17). Thus, additional evidence of the clinical significance of the E709_T710delins mutation needs to be explored.

For uncommon *EGFR* mutations, although the data from prospective clinical trials are insufficient because of the low frequency and diversity of such mutations, some cases harboring uncommon *EGFR* mutations have been reported with effective treatment by EGFR TKIs. It has been reported that the major uncommon *EGFR* mutations, including G719X, S768I, and L861Q are more sensitive to afatinib and osimertinib, compared to first-generation EGFR TKIs (18). Therefore, afatinib or osimertinib have been suggested as possible first-line treatment options for major uncommon *EGFR* mutations (19). However, patients with uncommon mutations that co-occurred with common *EGFR* mutations exhibited responses to first-generation EGFR TKIs (20). Limited clinical data and our analyses suggest that other rare *EGFR* mutations, including E709X, L747P/S, Del18 mutations, and some exon 19 insertion-deletions are more sensitive to afatinib or osimertinib than gefitinib or erlotinib (**Table 2**). In particular, NSCLC patients with compound *EGFR* mutations involving T790M exhibit good responses to osimertinib compared to NSCLC patients with other mutations (19).

In summary, this study reported a lung adenocarcinoma patient who harbored an *EGFR* E709_T710delinsD mutation and received the second-generation EGFR TKI, afatinib, as the first-line therapy. Unexpectedly, lung lesion shrinkage lasted 7 months and the patient achieved a PFS of up to 23 months. Following the development of afatinib-resistance, almonertinib was administered and achieved a SD. Thus, this case detailed a reliable treatment option for NSCLC patients harboring a rare *EGFR* exon 18 deletion-insertion mutation.

## Data Availability Statement

The original contributions presented in the study are included in the article/supplementary files. Further inquiries can be directed to the corresponding author.

## Ethics Statement

The studies involving human participants were reviewed and approved by Ethical committee of Jiangsu Province Hospital of Traditional Chinese Medicine. The patients/participants provided their written informed consent to participate in this study.

## Author Contributions

Conception and design: LZ, LL, and YW. Collection and assembly of data: YW, YG, and YC. Manuscript writing and revising: All authors. All authors contributed to the article and approved the submitted version.

## Conflict of Interest

YC and YG are employees of Nanjing Geneseeq Technology Inc., China.

The remaining authors declare that the research was conducted in the absence of any commercial or financial relationships that could be construed as a potential conflict of interest.
